# Distinct Host-Specific Bacterial Assemblages in Four Congeneric *Pocillopora* Corals Reveal a Minimal Core Microbiome and Probiotic Partitioning

**DOI:** 10.3390/microorganisms13092083

**Published:** 2025-09-06

**Authors:** Chenghao Chen, Shuailiang Xu, Maosen Shangguan, Meng Wang, Xiaofei Xiong

**Affiliations:** 1 Nansha Islands Coral Reef Ecosystem National Observation and Research Station, Guangzhou 510300, China; hancy_chen@163.com (C.C.); xushuailiang@gmail.com (S.X.); sgms@foxmail.com (M.S.); wangmeng2831@163.com (M.W.); 2 South China Sea Ecological Center, Ministry of Natural Resources, Guangzhou 510300, China

**Keywords:** coral, bacterial composition, 16S rRNA gene sequencing, *Pocillopora eydouxi*, *Pocillopora meandrina*, *Pocillopora verrucosa*, *Pocillopora woodjonesi*

## Abstract

Coral reefs, essential yet increasingly threatened marine ecosystems, rely on coral–microbiome symbioses for resilience against environmental stressors. This study investigates host-specific influences on bacterial communities in four *Pocillopora* species (*Pocillopora eydouxi*, *Pocillopora meandrina*, *Pocillopora verrucosa*, and *Pocillopora woodjonesi*) from the South China Sea. Using Illumina-based 16S rRNA gene sequencing, we analyzed microbiome structures, identified core taxa, and predicted metabolic functions. Results revealed that bacterial composition differed significantly among coral hosts, despite their shared habitat. *P. eydouxi* exhibited the highest bacterial richness and Shannon index, contrasting with minimal values in *P. woodjonesi*. A conserved core microbiome of 32 ASVs (1.1% of total ASVs), dominated by *Gammaproteobacteria*, was shared across all coral species. Host-specific enrichment of probiotic bacteria (*Psychrobacter* in *P. eydouxi* and *Exiguobacterium* in *P. meandrina*) and pathogenic taxa (e.g., *Acinetobacter*) was also observed. Functional prediction indicated conserved metabolic pathways across species, particularly amino acid and carbohydrate metabolism. These findings highlight host phylogeny as one of the primary determinants of microbiome assembly, providing critical insights into coral conservation strategies.

## 1. Introduction

Coral reefs, vital for coastal protection, seafood provision, biodiversity maintenance, and medicinal exploration, are among the most biodiverse and economically valuable marine ecosystems [1]. Global degradation of coral reefs has primarily resulted from human activities, exacerbated in recent years by climate warming [2], ocean acidification [3], and pollution pressures [4]. The frequency and intensity of mass coral bleaching events have significantly increased, becoming the foremost threat to coral reef health [5]. Elucidating the mechanisms underlying coral adaptation and environmental stress tolerance is critical for predicting the future of coral reefs and designing effective conservation strategies.

Corals are complex holobionts comprising *Symbiodiniaceae*, bacteria, archaea, and viruses, rather than autonomous biological entities [6]. Their health is closely linked to the stability of coral–microbiome symbioses, which play a pivotal role in marine biodiversity [7]. Research demonstrates that microorganisms associated with corals contribute to host health by engaging in critical processes such as nitrogen fixation, carbon cycling, and pathogen defense. Nitrogen-fixing bacteria associated with *Pocillopora damicornis* modulate nitrogen levels and mitigate bleaching effects [8], while bioactive compounds from beneficial bacteria of the coral *Porites lutea* exhibit remarkable antagonism against various coral pathogens [9]. Recent studies suggest that microbiome functions depend on dynamic network interactions rather than the activity of individual taxa [10,11]. A reduction in host biodiversity significantly alters coral microbial community structures, causing scleractinian corals to shift from active “maintenance-type” or “acquisition-type” strategies to passive defense mechanisms [6]. The disruption of this microbial network directly compromises coral resilience to environmental stressors.

*Pocillopora*, a predominant reef-building coral genus in Indo-Pacific ecosystems, serves as a key model for studying coral adaptability due to its broad geographic distribution, environmental sensitivity, and interspecies coexistence [12,13]. For example, *P. meandrina* exhibits consistent growth rates within a temperature range of 26.1–28.8 °C, indicating thermal resilience and host-specific microbiota [14]. These corals are widely distributed from tropical to subtropical regions, including the South China Sea, and can grow more than 20 cm in width, covered by various types of verrucae [15]. Notably, bacterial communities associated with *Pocillopora* show habitat specificity compared to surrounding seawater microbes, and their spatial clustering patterns reflect adaptations to localized environmental stressors [16]. *Pocillopora acuta* colonies near anthropogenically impacted sites exhibit significant variations in microbial diversity compared to adjacent less disturbed habitats, highlighting the influence of habitat conditions on coral-associated bacterial communities [17]. Previous studies have confirmed that *Pocillopora* adaptability arises from symbiotic partnerships with *Symbiodiniaceae* and bacteria, collectively facilitating responses to environmental variability. Environmental conditions drive compositional shifts in *Symbiodiniaceae* communities, allowing coral adaptation through modulation of sub-clade abundances [8]. In contrast, research on *P. damicornis* under varying pCO_2_ conditions revealed no significant changes in bacterial community composition, suggesting that ocean acidification may not significantly affect this species’ microbiome [3]. Furthermore, antibiotic disruption of native coral–microbiome symbiosis compromises the fitness of the *Pocillopora* holobiont through diminished oxygen consumption and induced immune responses, underscoring the essential role of bacterial associates in maintaining *Pocillopora* coral health [18]. While geographic distribution and environmental adaptations of *Pocillopora* species have been extensively documented [19], understanding remains limited regarding microbiome differences among congeneric species and the relative influences of species-specific versus environmental factors.

This study investigates host-specific bacterial assemblages in four *Pocillopora* species (*P. eydouxi*, *P. meandrina*, *P. verrucosa*, and *P. woodjonesi*) from the Nansha coral reefs in the South China Sea. Bacterial composition and diversity were characterized using high-throughput Illumina 16S rRNA gene sequencing. A comparative analysis of microbiome structures among the four species was conducted. Furthermore, core microbiome composition and inferred functional profiles of *Pocillopora*-associated bacterial communities were compared to clarify their potential ecological roles in coral reefs. By elucidating similarities and differences in microbial assemblages across distinct host backgrounds, our findings reveal how host identity shapes bacterial partnerships, providing new insights to inform coral reef conservation strategies.

## 2. Materials and Methods

### 2.1. Sample Collection

**Figure 1:**
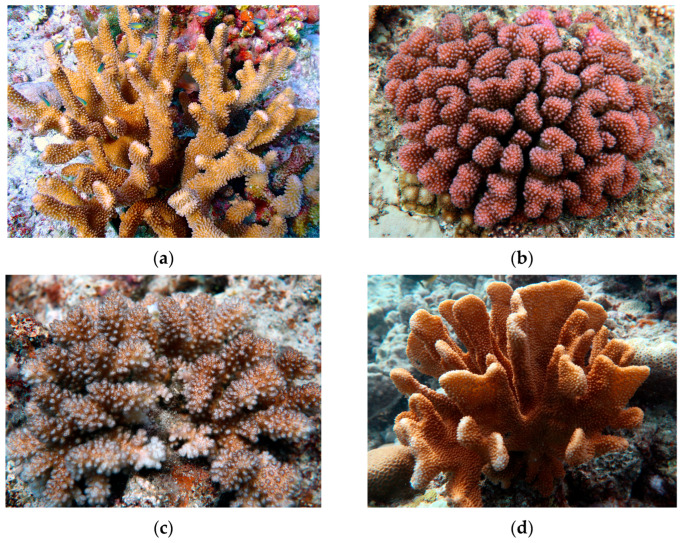
Photographs of the *Pocillopora* coral species from Nansha coral reefs: (**a**) *P. eydouxi*; (**b**) *P. meandrina*; (**c**) *P. verrucosa*; (**d**) *P. woodjonesi*.

Four *Pocillopora* coral samples were collected by SCUBA diving from the Nansha coral reefs in the South China Sea in June 2022 for symbiotic bacteria analysis. Four coral species (*P. eydouxi*, *P. meandrina*, *P. verrucosa*, and *P. woodjonesi)* were identified based on ecological characteristics and skeletal morphology (Figure 1). Prior to collection, all coral colonies were visually assessed for signs of disease or stress. Only colonies displaying normal pigmentation, full tissue coverage (no exposed skeleton), and no visible signs of bleaching, algal overgrowth, or lesions were selected for sampling. Three replicate samples (5–10 cm^2^ each) for each species were obtained within a 20 × 20 m area at depths ranging from 4 to 6 m. Coral surfaces were triple-rinsed with sterile seawater (salinity: 35‰), transferred to sterile sample bags, flash-frozen immediately in liquid nitrogen, and stored at −80 °C.

### 2.2. Genomic DNA Isolation and PCR Amplification

Genomic DNA from coral-associated bacteria was isolated (5–10 cm^2^ coral tissue per sample) using the E.Z.N.A.^®^ soil DNA Kit (Omega Bio-tek, Norcross, GA, USA), following the manufacturer’s instructions. DNA integrity and concentration were evaluated using 1.0% agarose gel electrophoresis and a Thermo Scientific spectrophotometer, with samples subsequently storage at −80 °C. The amplification of the V3–V4 region of the bacterial 16S rRNA gene employed primers 338F (ACTCCTACGGGAGGCAGCAG) and 806R (GGACTACHVGGGTWTCTAAT) [15]. The 20 µL reaction mixture comprised the following:

4 µL 5× Fast Pfu buffer

2 µL 2.5 mM dNTPs

0.8 µL each primer (5 µM)

0.4 µL Fast Pfu polymerase

0.2 µL bovine serum albumin (BSA)

10 ng of template DNA

ddH_2_O to 20 µL final volume.

Thermal cycling program: 95 °C for 3 min (initial denaturation); 29 cycles of 95 °C/30 s, 55 °C/30 s, and 72 °C/45 s; 72 °C for 10 min (final extension); and holding at 4 °C. Amplicons were electrophoresed on 2% agarose gels, purified with the AxyPrep DNA Gel Extraction Kit, and quantified via Quantus™ Fluorometer (Promega, Madison, WI, USA).

### 2.3. High-Throughput Sequencing

Following standardized protocols from Majorbio Bio-Pharm Technology Co., Ltd. (Shanghai, China), purified amplicons were combined at equimolar concentrations and subjected to paired-end sequencing (2 × 300 bp) on an Illumina MiSeq PE300 platform.

### 2.4. Amplicon Sequence Processing

Raw sequencing reads were demultiplexed, quality-filtered using fastp (version 0.19.6) [20], and merged using FLASH (version 1.2.11) [21]. High-quality reads were denoised into amplicon sequence variants (ASVs) at single-nucleotide resolution using the DADA2 [22] plugin within the Qiime2 pipeline (version 2020.2), with default error profiles. To standardize sequencing depth for diversity analyses, sequences were rarefied to 29,943 reads per sample. ASVs were taxonomically classified using the Naive Bayes consensus taxonomy classifier in Qiime2 with the SILVA 16S rRNA database (version 138). All interpretations and conclusions in this study are based on the SILVA taxonomic framework. Putative functional prediction was carried out using PICRUSt2 [23] based on representative ASV sequences. This analysis leverages the Kyoto Encyclopedia of Genes and Genomes (KEGG) database, assigning KEGG Orthology identifiers to putative genes and pathways.

### 2.5. Statistical Analysis

Bioinformatic analyses of coral microbiota were conducted on the Majorbio Cloud Platform (https://cloud.majorbio.com (accessed on 12 January 2023)). Alpha diversity (observed ASVs, Chao, Shannon, Simpson, Coverage, and phylogenetic diversity [Pd]) and rarefaction curves were generated with Mothur (version 1.30.1). Weighted Unifrac dissimilarity-based non-metric multidimensional scaling (NMDS) via the vegan (version 2.5–3) package was used to quantify beta diversity. The permutational multivariate analysis of variance (PERMANOVA) was employed to assess the proportion of variation and its statistical significance between groups. LEfSe analysis identified differentially abundant bacterial taxa (phylum to genus) between groups (LDA > 4; *p* < 0.05).

## 3. Results

### 3.1. Sequencing Information and Bacterial Diversity Analysis

The bacterial read counts obtained from twelve *Pocillopora* coral samples ranged from 29,943 to 39,402, resulting in 2963 ASVs at a 100% threshold (i.e., sequences present in all samples within each group). The average ASV sequence length in each sample ranged from 420 bp to 425 bp. Despite originating from the same genus and reef, significant variations in bacterial diversity and abundance were observed among coral species. Specifically, samples from *P. eydouxi* exhibited higher richness and Shannon indices compared to other species. Simpson indices across samples ranged from 0.08 to 0.36. Additional diversity metrics, including Sobs, Chao, and Pd, are provided in Table 1. Each sample library showed community coverage exceeding 99.8%, consistent with rarefaction analysis results (Figure S1). Thus, sequencing adequately represented the coral-associated bacterial community.

**Figure 2:**
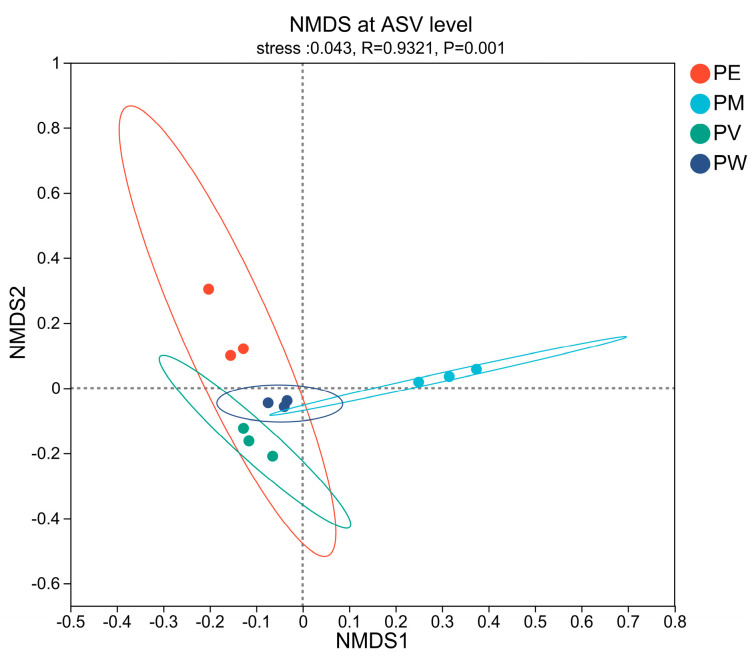
NMDS analysis at the ASV level based on weighted Unifrac dissimilarity among coral samples.

To examine variations in bacterial communities among coral species, NMDS analysis using weighted Unifrac similarity was performed (stress = 0.043, R = 0.932, *p* = 0.001). The NMDS plot (Figure 2) revealed clear distinctions in bacterial composition across species. PERMANOVA (R^2^ = 0.696, *p* = 0.001) confirmed that coral-associated bacterial community compositions significantly differed among the samples collected from the four *Pocillopora* coral species.

### 3.2. Structure of Coral-Associated Bacterial Communities

**Figure 3:**
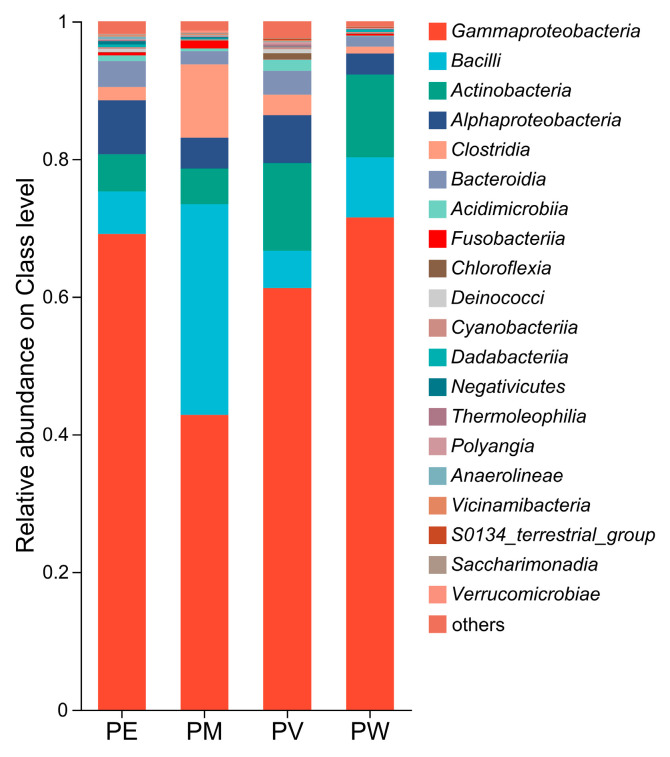
Relative abundances of bacterial classes.

**Figure 4:**
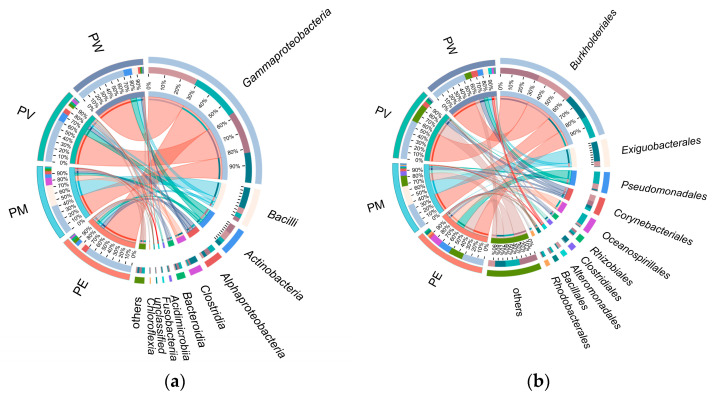

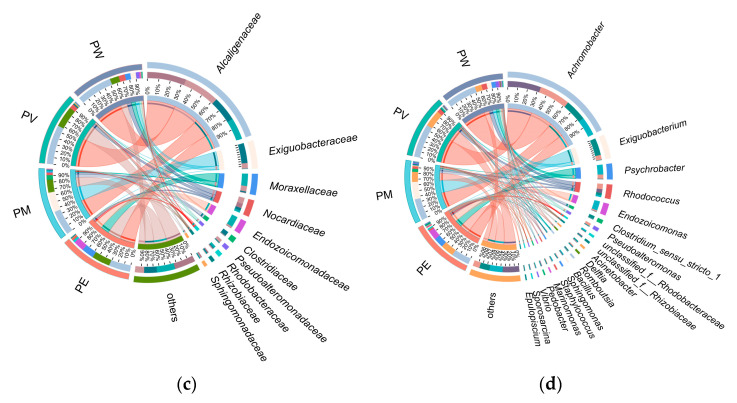
Taxonomic distribution of bacterial communities based on sequence analysis: (**a**) class level; (**b**) order level; (**c**) family level; (**d**) genus level.

Overall, twenty bacterial classes exhibited an average relative abundance ≥0.1% (Figure 3). *Gammaproteobacteria* was overwhelmingly predominant (average relative abundance: 61.3%; Figure 4a), followed by *Bacilli* (12.8%), *Actinomycetota* (8.8%), *Alphaproteobacteria* (5.5%), *Clostridia* (4.3%), and *Bacteroidia* (2.8%). Other classes showed relatively low abundance (<2.0%). At the order level, *Burkholderiales* displayed the highest abundance, ranging from 32% to 58% across groups (Figure 4b). The second most abundant orders differed among groups: *Corynebacteriales* (9%) in PW, *Corynebacteriales* (10.16%) in PV, *Exiguobacterales* (8.81%) in PM, and *Pseudomonadales* in PE. As shown in Figure 4b,c, relative abundances at the family level mirrored those observed at the order level. Apart from *Alcaligenaceae*, other families (*Exiguobacteraceae*, *Moraxellaceae*, *Nocardiaceae*, and *Endozoicomonadaceae*) varied in their contributions to bacterial communities across the coral species.

The analysis revealed distinct profiles of dominant bacterial genera across the different coral species. The five most abundant genera overall were *Achromobacter*, *Exiguobacterium*, *Psychrobacter*, *Rhodococcus*, and *Endozoicomonas*. Notably, the abundance of the top 20 genera were higher in the PE group compared to the others. Within specific coral groups, *Achromobacter* was the dominant genus in the PV group. The PM group was predominantly characterized by *Exiguobacterium*, *Clostridium*, and *Acinetobacter*. In contrast, *Psychrobacter* and *Endozoicomonas* were significantly more abundant in the PW group (Figure 4d).

**Figure 5:**
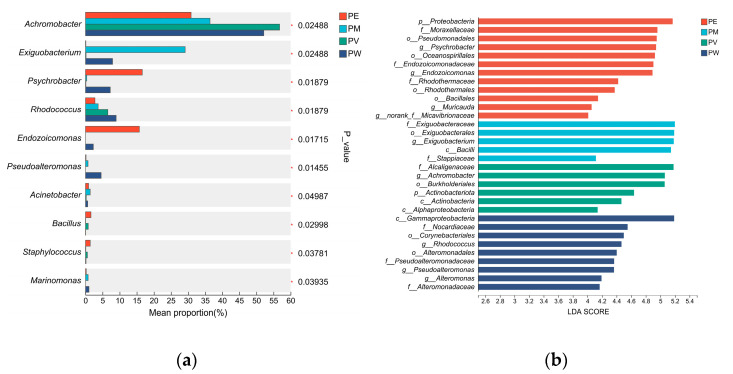
Differential analysis of bacterial communities: (**a**) Wilcoxon rank-sum test showing significant differences at the genus level; (**b**) LEfSe discriminant analysis (LDA scores).

Bacterial composition (genus level) differed significantly among coral groups (Wilcoxon rank-sum test, *p* < 0.05; Figure 5a). *Achromobacter* (*p* = 0.025) exhibited significantly higher relative abundance in the PV group. Moreover, the PE group demonstrated significantly higher abundances of *Psychrobacter* (*p* = 0.019), *Endozoicomonas* (*p* = 0.017), *Bacillus* (*p* = 0.030), and *Staphylococcus* (*p* = 0.039) compared with other groups. Similarly, the PM group showed notably higher relative abundances of *Exiguobacterium* (*p* = 0.025) and *Acinetobacter* (*p* = 0.049) compared to other groups. Furthermore, the PW group exhibited significantly elevated abundances of *Rhodococcus* (*p* = 0.015), *Pseudoalteromonas* (*p* = 0.019), and *Marinomonas* (*p* = 0.039) relative to other coral groups. Figure 5b illustrates LDA values for significantly different taxa, visually representing distinctive marker taxa across groups, ranging from phylum to genus. Specifically, at the phylum level, the PE and PV groups were enriched in *Actinomycetota*. At the order level, the PE group was enriched in *Rhodothermales* and *Bacillales*, the PM group in *Exiguobacterales*, and the PV group in *Burkholderiales*. The PW group was enriched in the class *Gammaproteobacteria*.

### 3.3. Composition of the Core Microbiome

**Figure 6:**
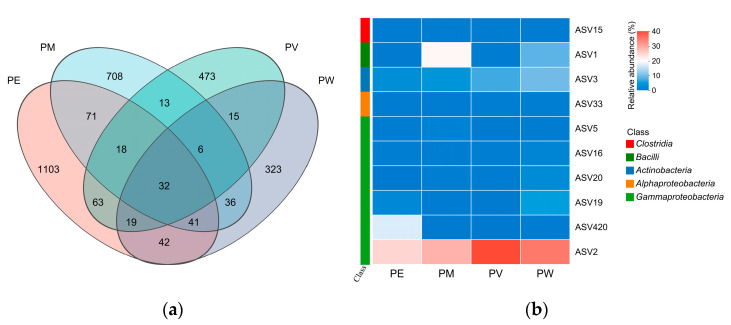
Composition analysis of the core microbiome: (**a**) Venn diagram of bacterial ASVs associated with *Pocillopora* corals; (**b**) Heatmap illustrating core ASV abundances across *Pocillopora* coral species.

Among the total 2963 ASVs identified, the PE, PM, PV, and PW groups contributed 1389, 925, 639, and 514 ASVs, respectively. Only 32 ASVs were shared across the four groups, constituting 1.1% of the total ASVs. These shared ASVs accounted for 2.3%, 3.5%, 5.0%, and 6.2% of the total ASVs in the PE, PM, PV, and PW groups, respectively. Conversely, unique ASVs represented from 10.9% to 37.2% of the total ASVs identified in the coral samples (Figure 6a).

Taxonomic affiliations of core ASVs were determined using online BLAST analysis against reference databases (Table S1). The core microbiome, comprising 10 ASVs, represented less than 1.2% of the total ASVs per coral (average of 867 ASVs), but exhibited notably high relative abundances across all four groups (58.26–79.34%). Variability in relative abundance was considerable among groups. Predominantly, core ASVs belonged to the class *Gammaproteobacteria*, with ASV2 consistently the most abundant across all groups (30.78–56.71%). Differences in core ASVs were also observed among coral species. Specifically, six core ASVs in the PW group (ASV1, ASV2, ASV3, ASV16, ASV19, and ASV20) each had relative abundances exceeding 1%. The PE group showed significantly higher abundances of ASV420 compared with other groups. Additionally, ASV1 and ASV5 were more prevalent in the PM group, while the PV group predominantly harbored ASV2 and ASV3, each with relative abundances exceeding 5% (Figure 6b).

### 3.4. Predicted Functional Profiles in Pocillopora Corals

**Figure 7:**
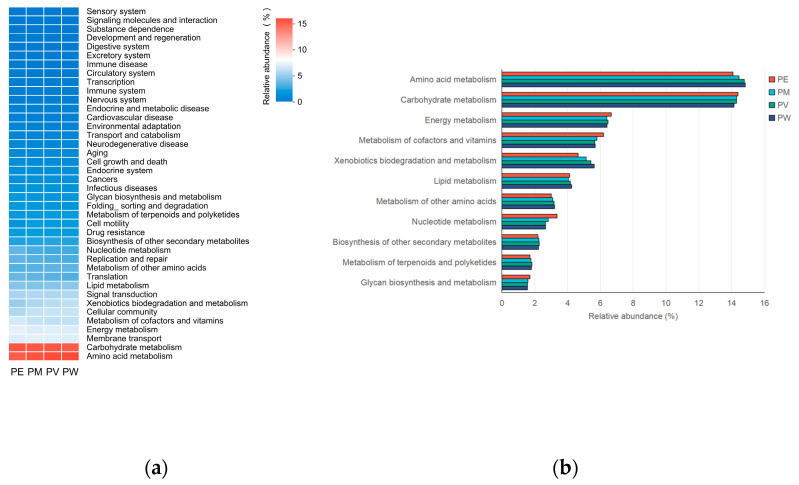
Functional predictions of bacterial communities: (**a**) Dominant gene families for each group; (**b**) Relative abundance of predicted metabolic pathways across groups.

Putative functional profiles of the coral-associated bacterial communities were predicted using PICRUSt2 based on 16S rRNA gene sequencing data. It should be noted that this method provides inferred functional profiles based on phylogenetic predictions and reference genomes. This analysis predicted abundances for 40 gene families within KEGG level 2 pathways (Figure 7a). Analysis of these predicted pathways revealed no significant differences among bacterial communities of the four *Pocillopora* corals. The KEGG category “Metabolism”, which encompasses processes such as carbohydrate, energy, amino acid, and nucleotide metabolism, was predicted to be the predominant functional category (61.6–62.4% of putative genes). This category was further subdivided into eleven metabolic subcategories (Figure 7b). These subcategories exhibited similar relative abundances across coral species. Notably, carbohydrate metabolism and amino acid metabolism were predicted to have the highest relative abundances (each >14%), while other metabolic categories had predicted abundances below 7%. Additionally, hypothetical microbial functions were inferred using KEGG-based KO annotations, summarized as the top 30 predicted metabolic pathways (Table S2). These functional predictions represent testable hypotheses regarding potential microbial metabolism within the coral holobiont, rather than direct evidence of biochemical activity.

## 4. Discussion

The coral microbiome is essential for maintaining ecological functions and overall reef health [6]. The intricate relationships between scleractinian corals and their bacterial communities have emerged as critical factors in coral reef ecology. Previous studies have demonstrated diverse bacterial compositions and complex functionalities within coral-associated microbiota [18,24]. Coral-associated bacteria contribute significantly to nitrogen fixation [25], organic matter mineralization [11], and vitamin synthesis [8], thereby enhancing coral energy metabolism and nutrient availability. Additionally, these bacterial communities can enhance coral resilience to environmental stressors, including heat stress [26] and ocean acidification [27], thereby mitigating the risk of coral bleaching. Conversely, coral bleaching events can promote the proliferation of pathogenic bacteria, destabilizing the coral–microbial symbiosis and compromising coral health [28]. The present study characterizes bacterial communities in four ecologically significant *Pocillopora* species, aiming to advance the understanding of coral-associated bacterial community structures and functions in reef ecosystems.

### 4.1. Bacterial Diversity in Pocillopora Corals

The bacterial α-diversity index in *Pocillopora* corals positively correlates with the complexity of their microbial interaction networks. Increased network complexity typically indicates stronger interdependencies among individual microorganisms [10,29]. However, numerous studies have demonstrated that environmental stressors, including elevated temperatures [26,30], acidification [31], and eutrophication [4], can increase bacterial alpha diversity in corals while simultaneously compromising microbial community stability. In this study, samples from *P. eydouxi* exhibited the highest mean Chao richness and Shannon diversity indices, followed by *P. damicornis* and *P. verrucosa*, with *P. woodjonesi* showing the lowest. These results suggest that *P. eydouxi* may possess a less stable microbial community compared to other species. Because corals represent open microbial systems, increased bacterial α-diversity typically signals reduced capacity to regulate microbial communities and resist pathogen colonization. The elevated bacterial α-diversity observed in corals is primarily attributed to invasions by opportunistic and pathogenic bacteria from the surrounding environment [29]. Consequently, the positive correlation between alpha diversity and microbial interaction network complexity observed in this study may indicate decreased microbial community stability. Environmental stress can eliminate key microbial community members, impair interactions among microorganisms, and significantly weaken the resilience of the microbial community to external stressors [32,33,34]. Thus, *P. eydouxi* characterized by higher bacterial α-diversity, may be more susceptible to declines in symbiotic stability and health under environmental stress, whereas *P. woodjonesi* might display greater adaptability.

As illustrated in Figure 3, the dominance of *Proteobacteria* (syn. *Pseudomonadota*) represented by multiple genera, and *Firmicutes* (syn. *Bacillota*), particularly *Exiguobacterium* and *Clostridium*, aligns with previous studies of Indo-Pacific corals, confirming their ubiquity in coral holobionts [35,36,37]. This study identified a core microbiome shared across the four coral species, although genus-level compositions (e.g., *Achromobacter*, *Exiguobacterium*, *Psychrobacter*, *Rhodococcus*, *Endozoicomonas*, and *Pseudoalteromonas*) differed by host species (Figure 5a). Previous research has shown that differences in mucus composition may influence bacterial community structures in coral reef habitats [16,38]. For instance, the mucus-associated bacterial composition of *P. damicornis* was dominated by *Gammaproteobacteria*, Cytophagia, and Actinomycetia, significantly differing from that of *Stylophora pistillata* [16]. Additionally, immunological and microbiological responses in corals are highly localized [39], suggesting that phylogenetic variations within *Pocillopora* might select for distinct microbial partners through species-specific mucus composition, immune responses, or microhabitat conditions [26]. Notably, the absence of certain coral-associated bacteria such as *Ralstonia*, frequently reported in other studies, merits attention [28,40]. This discrepancy is likely influenced by ecological determinants. It is important to emphasize that sampled corals in this study were not affected by bleaching, as no bleaching event occurred in the sampling area. Interestingly, research on corals from the coastal regions of Hainan’s Luhuitou Peninsula reported a higher abundance of *Ralstonia* in non-bleached corals compared to bleached ones [7]. The fact that *Ralstonia* was undetected in this non-bleached environment suggests that factors other than thermal stress may play a more decisive role.

### 4.2. Core Bacterial Communities in Pocillopora Corals

Bacterial communities associated with corals demonstrate functional versatility and adaptive capacity across developmental stages and environmental gradients. These symbionts critically influence coral health, nutrient dynamics, and host development. Among the four *Pocillopora* species studied, ASV2 (*Achromobacter*, phylum *Proteobacteria*) represents the largest proportion of the core microbiome. Similar findings were reported in *P. damicornis* populations from the northern South China Sea [41]. Prior studies have reported elevated *Achromobacter* abundance in healthy versus bleached corals [42], highlighting its significance as a key bacterium involved in biological nitrogen fixation [43].

*Proteobacteria* constitute a fundamental component of coral microbiota, underpinning reef ecosystem functionality. However, the relative abundances of *Psychrobacter* (ASV19), *Pseudoalteromonas* (ASV20), and *Endozoicomonas* (ASV420) vary markedly among *Pocillopora* species. *Endozoicomonas* exhibits unique metabolic functions, notably synthesizing amino acids and vitamins unavailable from the coral host [44], and preventing mitochondrial dysfunction under environmental stress [45]. Similarly, *Psychrobacter* and *Pseudoalteromonas* exhibit strong antibacterial activity against coral pathogens [46,47], beneficially supporting coral host health. The variations in probiotic bacterial abundance observed across the four *Pocillopora* species suggest that coral hosts possess species-specific physiological mechanisms for selecting probiotic symbionts, indicating highly flexible and dynamic bacterial communities. Notably, core ASV1, closely related to *Exiguobacterium*, exhibited significantly higher relative abundance in the PM and PW groups, compared to the PE and PV groups. Previous studies demonstrated that *Exiguobacterium* produces small, ethanol-soluble molecules capable of inhibiting the induction of catabolic enzymes in pathogens [48]. The diverse metabolic capabilities and environmental stress resistance exhibited by *Exiguobacterium* contribute to its cosmopolitan distribution [49]. Additionally, *Rhodococcus* (ASV3), belonging to the phylum *Actinomycetota*, has shown antibacterial activity against *Bacillus* [50]. The varying abundances of probiotic bacteria associated with different *Pocillopora* species suggest that further investigation is required to understand the synergistic effects of different probiotic compositions on coral host health and environmental adaptation.

Using in vitro pathogenicity assays, Rahmi et al. demonstrated that *Acinetobacter* sp. can trigger coral brown band disease [51], characterized by a distinctive brown band demarcating healthy tissue from the exposed skeleton [52]. This disease causes tissue necrosis and mortality in coral colonies, leading to significant reductions in coral cover and biodiversity [53]. Notably, although *Acinetobacter* (ASV5) was identified as a core member of the *Pocillopora* microbiome in our study, field observations revealed no pathological abnormalities in the coral holobiont, contrasting markedly with previous findings. This suggests that taxa with pathogenic potential may exist as harmless commensals within a stable and healthy core microbiome. The overall microbial community structure and function are therefore critical for maintaining beneficial host–microbe relationships and suppressing virulence expression in potential pathogens. Nevertheless, this balance is fragile; intensified global climate change and anthropogenic disturbances may disrupt the core microbial community, reducing environmental resilience and increasing coral susceptibility to infectious diseases.

### 4.3. Functional Profiles in Pocillopora Corals

The metabolic functions of coral-associated bacteria are essential for coral health and reef ecosystem resilience, mediated by interconnected mechanisms involving nutrient provision, environmental adaptation, and pathogen defense [8]. Putative functional predictions indicated a predominance of metabolic pathways within coral-associated bacterial communities, particularly those linked to amino acid and carbohydrate metabolism. These findings are consistent with previous reports and highlight the potential prevalence of core metabolic processes crucial for the survival of coral-associated microbiota [54]. Amino acids and carbohydrates are essential for biosynthesis, growth, and nitrogen recycling, thereby regulating interactions between *Symbiodiniaceae* and coral-associated bacteria [55]. Previous studies demonstrated that amino acid metabolic trophallaxis between *Symbiodiniaceae* and bacteria such as *Pseudoalteromonas* and *Bacillus* significantly enhances survival and growth in nitrogen-limited coral reef environments [56]. Such metabolic functions are especially critical for nutrient cycling and stress-tolerance within the coral holobiont.

In addition to predictions of metabolic functions, KEGG analysis inferred relatively high abundances of motility genes associated with membrane transport, cellular community, and signal transduction (Figure 7a). These predicted functions potentially facilitate bacterial association with coral hosts under environmental stress conditions [57]. Previous metagenomic studies confirmed enrichment of genes involved in membrane transport, carbohydrate metabolism, amino acid metabolism, bacterial chemotaxis, and cell motility in bleached *Acropora cytherea* and *Goniastrea minuta* compared to healthy corals [54]. However, a contradictory KEGG-based analysis by Meiting et al. reported no significant differences in predicted bacterial functional profiles between non-bleached and bleached corals (*A. digitifera*, *G. fascicularis*, and *Porites pukoensis*) [7]. This discrepancy suggests that bacteria may adapt to stressors within specific hosts by maintaining stable metabolic potential, thus potentially enabling coral recovery post-bleaching events. Meanwhile, stress-induced alterations in coral microbiota diversity and abundance may profoundly affect microbial metabolic functions, potentially shifting coral holobiont health status [58]. For instance, ocean acidification reportedly induces functional shifts in coral microbiomes, redirecting microbial carbohydrate metabolic pathways from energy production toward maintaining cellular membrane and wall integrity [59]. Additionally, coral-associated bacterial communities are recognized as key determinants of coral disease resistance, particularly concerning energy metabolism and immune response pathways [60]. This inferred potential may explain why *Acinetobacter* (ASV5), despite being a core microbiome member, does not adversely affect coral health.

The predictions presented in this study, generated from 16S rRNA gene data using PICRUSt2, infer metabolic potential based on taxonomic profiles and reference genomes. Therefore, the functional profiles discussed here represent the potential of the microbial community inferred from its taxonomic composition. In future studies, we plan to undertake metagenomic and metabolomic studies to experimentally validate these inferred functional capabilities.

## 5. Conclusions

This investigation establishes that host phylogeny is a primary determinant of bacterial community assembly in *Pocillopora* corals from the South China Sea, despite shared environmental conditions. Significant interspecies differences were observed in microbiome diversity and composition: *P. eydouxi* exhibited the highest bacterial richness and Shannon diversity, while *P. woodjonesi* displayed the lowest. A minimal core microbiome consisting of 32 ASVs (predominantly *Gammaproteobacteria*) was conserved across species, representing only 1.1% of total ASVs yet accounting for 58.26–79.34% of the relative abundance. Host-specific enrichment of bacterial taxa was evident, including both potentially beneficial bacteria and taxa with opportunistic potential. Functional predictions revealed conserved metabolic profiles primarily associated with amino acid and carbohydrate metabolism, which may play critical roles in nutrient cycling and stress tolerance. These findings suggest that *Pocillopora* species may employ species-specific mechanisms to select their bacterial associates, resulting in highly adaptable microbial communities. Despite substantial taxonomic variation, no significant differences were found in core metabolic functions among coral species. The host-specific beneficial bacterial profiles observed in this study may inform future research on targeted intervention strategies. Future studies should explore the dynamic functional responses of host-adapted microbiomes under environmental stress to elucidate mechanisms of functional plasticity and to better define thresholds for coral resilience.

## Figures and Tables

**Table 1:** Sequencing information and alpha diversity estimates for coral-associated bacteria.

Group	Sample	Effective Reads	Sobs	Chao	Shannon	Simpson	Coverage	Pd
PE	*P. eydouxi* 1	35,139	432	434.33	3.40	0.17	99.94%	62.19
	*P. eydouxi* 2	35,162	687	708.21	4.00	0.08	99.75%	75.28
	*P. eydouxi* 3	32,683	546	550.52	3.52	0.16	99.90%	52.60
PM	*P. meandrina* 1	39,402	436	444.53	2.67	0.23	99.86%	49.53
	*P. meandrina* 2	29,943	400	405.43	3.05	0.19	99.91%	49.98
	*P. meandrina* 3	34,570	322	331.29	2.52	0.26	99.88%	40.82
PV	*P. verrucosa* 1	31,564	303	306.64	2.72	0.32	99.94%	32.03
	*P. verrucosa* 2	32,802	153	155.50	2.66	0.30	99.97%	23.19
	*P. verrucosa* 3	35,121	271	282.05	2.54	0.36	99.91%	26.40
PW	*P. woodjonesi* 1	36,094	157	158.17	2.01	0.32	99.97%	20.78
	*P. woodjonesi* 2	36,230	302	307.88	2.57	0.28	99.92%	31.51
	*P. woodjonesi* 3	32,354	148	148.38	2.40	0.28	99.99%	20.76

## Data Availability

The raw sequence data reported in this paper have been deposited in the Genome Sequence Archive (Genomics, Proteomics & Bioinformatics 2021) in the National Genomics Data Center (Nucleic Acids Res 2022), China National Center for Bioinformation/Beijing Institute of Genomics, Chinese Academy of Sciences (GSA: CRA029164) and are publicly accessible at https://ngdc.cncb.ac.cn/gsa (accessed on 25 August 2025). All original contributions presented in this study are included in the article and Supplementary Material. Further inquiries can be directed to the corresponding author.

## Supplementary Materials

The following supporting information can be downloaded at: https://www.mdpi.com/article/10.3390/microorganisms13092083/s1, Figure S1: Rarefaction curves: (a) Based on observed ASV number (Sobs), (b) Based on Shannon index; Table S1: Classification information of core ASVs associated with four Pocillopora corals; Table S2: Gene annotation and enriched pathways.

## Abbreviations

The following abbreviations are used in this manuscript:

ASV	Amplicon Sequence Variants
KEGG	Kyoto Encyclopedia of Genes and Genomes
NMDS	Non-metric Multidimensional Scaling
PERMANOVA	Permutational Multivariate Analysis of Variance
