# Supplementary material for: Distinct Host-Specific Bacterial Assemblages in Four Congeneric Pocillopora Corals Reveal a Minimal Core Microbiome and Probiotic Partitioning
Source: Microorganisms. 2025 Sep 6;13(9):2083. doi: 10.3390/microorganisms13092083 (PMC12472535; doi:10.3390/microorganisms13092083)
Supplement: Supplementary file 1 [file microorganisms-13-02083-s001.zip › microorganisms-3832818-supplementary.pdf]

Supplementary Materials

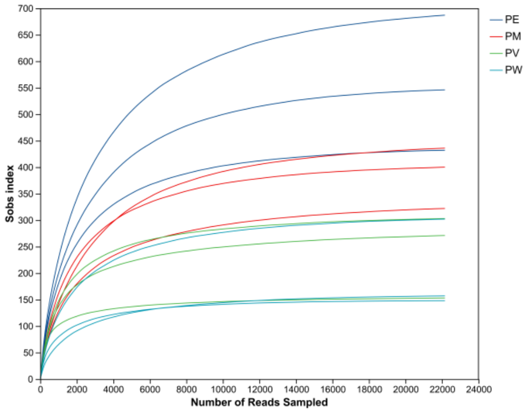

(**a**)

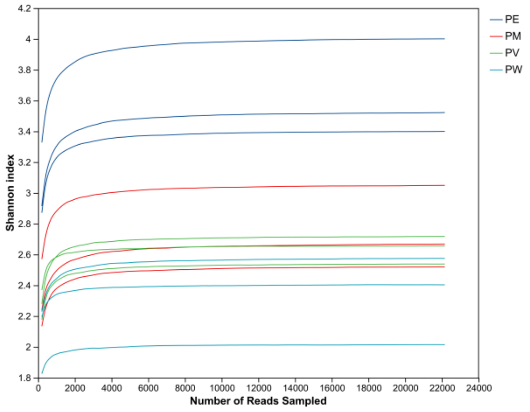

(**b**)

Supplementary Figure S1. Rarefaction curves: (a) Based on observed ASVs number (Sobs) ; (b) Based on Shannon index.

Supplementary Table S1. Classification information of core ASVs associated with four *Pocillopora* corals.

Entry	Classification information (SILVA version 138)			BLAST in NCBI (https://blast.ncbi.nlm.nih.gov/Blast.cgi)		
	**Phylum level**	**Class level**	**Genus level**	**Close match**	**accession number**	**Similarity**
ASV1	*Firmicutes*	*Bacilli*	*Exiguobacterium*	*Exiguobacterium aestuarii strain TF-16*	NR_043005.1	100%
ASV2	*Proteobacteria*	*Gammaproteobacteria*	*Achromobacter*	*Achromobacter deleyi strain LMG 3458*	NR_152014.1	99.77%
ASV3	*Actinomycetota*	*Actinomycetota*	*Rhodococcus*	*Rhodococcus qingshengii strain djl-6-2*	NR_115708.1	100%
ASV5	*Proteobacteria*	*Gammaproteobacteria*	*Acinetobacter*	*Acinetobacter lwoffii strain JCM 6840*	NR_113346.1	100%
ASV15	*Firmicutes*	*Clostridia*	*Romboutsia*	*Romboutsia sedimentorum strain LAM201*	NR_134800.1	100%
ASV16	*Proteobacteria*	*Gammaproteobacteria*	*Delftia*	*Delftia acidovorans strain LMG 1226*	NR_116139.1	100%
ASV19	*Proteobacteria*	*Gammaproteobacteria*	*Psychrobacter*	*Psychrobacter aestuarii strain SC35*	NR_116520.1	99.77%
ASV20	*Proteobacteria*	*Gammaproteobacteria*	*Pseudoalteromonas*	*Pseudoalteromonas gelatinilytica strain NH153*	NR_152003.1	100%
ASV33	*Proteobacteria*	*Alphaproteobacteria*	*Sphingomonas*	*Sphingomonas naasensis strain KIS18-15*	NR_133864.1	99.75%
ASV420	*Proteobacteria*	*Gammaproteobacteria*	*Endozoicomonas*	*Endozoicomonas atrinae strain WP70*	NR_134024.1	95.34%

Supplementary Table S2. Gene annotation and enriched pathways.

Entry	Symbol	Name	Pathway or Brite
K00059	fabG; OAR1	3-oxoacyl-acyl-carrier protein reductase	Fatty acid biosynthesis; Prodigiosin biosynthesis; Biotin metabolism; Metabolic pathways; Biosynthesis of secondary metabolites; Fatty acid metabolism; Biosynthesis of cofactors
K00626	ACAT; atoB	acetyl-CoA C-acetyltransferase	Fatty acid degradation; Valine, leucine and isoleucine degradation; Lysine degradation; Benzoate degradation; Tryptophan metabolism; Pyruvate metabolism; Glyoxylate and dicarboxylate metabolism; Butanoate metabolism; Other carbon fixation pathways; Terpenoid backbone biosynthesis; Metabolic pathways; Biosynthesis of secondary metabolites; Microbial metabolism in diverse environments; Carbon metabolism; Fatty acid metabolism; Two-component system; Fat digestion and absorption
K00799	GST; gst	glutathione S-transferase	glutathione metabolism; metabolism of xenobiotics by cytochrome P450; drug metabolism-cytochrome P450; drug metabolism-other enzymes; metabolic pathways; platinum drug resistance; longevity regulating pathway-worm; pathways in cancer; chemical carcinogenesis-DNA adducts; chemical carcinogenesis-receptor activation; chemical carcinogenesis-reactive oxygen species; hepatocellular carcinoma; fluid shear stress and atherosclerosis
K01652	E2.2.1.6L; ilvB; ilvG; ilvI	acetolactate synthase I/II/III large subunit	Valine, leucine and isoleucine biosynthesis; Butanoate metabolism; C5-Branched dibasic acid metabolism; Pantothenate and CoA biosynthesis; Metabolic pathways; Biosynthesis of secondary metabolites; 2-Oxocarboxylic acid metabolism; Biosynthesis of amino acids
K01714	dapA	4-hydroxy-tetrahydrodipicolinate synthase	Monobactam biosynthesis; Lysine biosynthesis;Metabolic pathways; Biosynthesis of secondary metabolites; Microbial metabolism in diverse environments; Biosynthesis of amino acids
K01897	ACSL, fadD	long-chain-fatty-acid---CoA ligase	Fatty acid biosynthesis; Fatty acid degradation; Metabolic pathways; Fatty acid metabolism; Quorum sensing; PPAR signaling pathway; Peroxisome; Ferroptosis; Thermogenesis; Adipocytokine signaling pathway
K01990	ABC-2.A	ABC-2 type transport system	ABC transporters

Entry	Symbol	Name	Pathway or Brite
		ATP-binding protein	
K01992	ABC-2.P	ABC-2 type transport system permease protein	ABC transporters
K01995	livG	branched-chain amino acid transport system ATP-binding protein	ABC transporters; Quorum sensing
K01996	livF	branched-chain amino acid transport system ATP-binding protein	ABC transporters; Quorum sensing
K01997	livH	branched-chain amino acid transport system permease protein	ABC transporters; Quorum sensing
K01998	livM	branched-chain amino acid transport system permease protein	ABC transporters; Quorum sensing
K01999	livK	branched-chain amino acid transport system substrate-binding protein	ABC transporters; Quorum sensing
K02014	TC.FEV.OM	iron complex outermembrane recepter protein	Other transporters
K02028	ABC.PA.A	polar amino acid transport system ATP-binding protein	ABC transporters
K02029	ABC.PA.P	polar amino acid transport system permease protein	ABC transporters
K02030	ABC.PA.S	polar amino acid transport system substrate-binding protein	ABC transporters
K02031	ddpD	peptide/nickel transport system ATP-binding protein	ABC transporters
K02032	ddpF	peptide/nickel transport system ATP-binding protein	ABC transporters
K02033	ABC.PE.P	peptide/nickel transport system permease protein	ABC transporters
K02034	ABC.PE.P1	peptide/nickel transport system permease protein	ABC transporters
K02035	ABC.PE.S	peptide/nickel transport system substrate-binding protein	ABC transporters
K02049	ABC.SN.A	NitT/TauT family transport system ATP-binding protein	ABC transporters
K02050	ABC.SN.P	NitT/TauT family transport system permease protein	ABC transporters
K02051	ABC.SN.S	NitT/TauT family transport system substrate-binding protein	ABC transporters
K03088	rpoE	RNA polymerase sigma-70 factor; ECF subfamily	transcription machinery (bacterial type)
K03406	mcp	methyl-accepting chemotaxis protein	Two-component system; Bacterial chemotaxis
K03719	lrp	Lrp/AsnC family transcriptional regulator, leucine-responsive regulatory protein	Transcription factors (Prokaryotic type)

Entry	Symbol	Name	Pathway or Brite
K07090	uncharacterized protein	uncharacterized protein	Function unknown
K07165	fecR	transmembrane sensor	Two-component system

Table information reference from https://www.genome.jp/kegg/
